# A mixture model for expression deconvolution from RNA-seq in heterogeneous tissues

**DOI:** 10.1186/1471-2105-14-S5-S11

**Published:** 2013-04-10

**Authors:** Yi Li, Xiaohui Xie

**Affiliations:** 1 Department of Computer Science, University of California, Irvine, CA, USA; 2 Institute for Genomics and Bioinformatics, University of California, Irvine, CA, USA; 3 Center for Machine Learning and Intelligent Systems, University of California, Irvine, CA, USA

## Abstract

**Background:**

RNA-seq, a next-generation sequencing based method for transcriptome analysis, is rapidly emerging as the method of choice for comprehensive transcript abundance estimation. The accuracy of RNA-seq can be highly impacted by the purity of samples. A prominent, outstanding problem in RNA-seq is how to estimate transcript abundances in heterogeneous tissues, where a sample is composed of more than one cell type and the inhomogeneity can substantially confound the transcript abundance estimation of each individual cell type. Although experimental methods have been proposed to dissect multiple distinct cell types, computationally "deconvoluting" heterogeneous tissues provides an attractive alternative, since it keeps the tissue sample as well as the subsequent molecular content yield intact.

**Results:**

Here we propose a probabilistic model-based approach, Transcript Estimation from Mixed Tissue samples (TEMT), to estimate the transcript abundances of each cell type of interest from RNA-seq data of heterogeneous tissue samples. TEMT incorporates positional and sequence-specific biases, and its online EM algorithm only requires a runtime proportional to the data size and a small constant memory. We test the proposed method on both simulation data and recently released ENCODE data, and show that TEMT significantly outperforms current state-of-the-art methods that do not take tissue heterogeneity into account. Currently, TEMT only resolves the tissue heterogeneity resulting from two cell types, but it can be extended to handle tissue heterogeneity resulting from multi cell types. TEMT is written in python, and is freely available at https://github.com/uci-cbcl/TEMT.

**Conclusions:**

The probabilistic model-based approach proposed here provides a new method for analyzing RNA-seq data from heterogeneous tissue samples. By applying the method to both simulation data and ENCODE data, we show that explicitly accounting for tissue heterogeneity can significantly improve the accuracy of transcript abundance estimation.

## Background

The rapidly advancing next-generation sequencing based transcriptome analysis tool, RNA-seq, provides a comprehensive and accurate method for analyzing the entire RNA components of the transcriptome [1]. The efficiency and sensitivity of RNA-seq make it a primary method for detecting alternatively-spliced forms and estimating their abundances [2,3]. However, estimating transcript abundances in heterogeneous tissues by RNA-seq remains an unsolved, outstanding problem because of the confounding effect from different cell types [4]. Many tissue samples from native environments are heterogeneous. For example, tumor samples are usually composed of tumor cells and surrounding normal cells [5]. Therefore, reads from an RNA-seq experiment of tumor samples will consist of contributions from both tumor and normal cells. Additionally, tumor tissues themselves are often heterogeneous, consisting of different subclones (e.g. breast cancer subtypes [6]), leading to even more complicated tissue environments.

Experimental methods have been proposed to address issues arising from contamination of different cell types, such as laser-capture microdissection [7], which allows dissection of morphologically distinguishable cell types. The mRNA content yield by this technology is consequently lowered, and needs to be compensated for, usually by molecular amplification. However, the nonlinearity induced by amplifying mRNA [8] has its own problems, and can make the expression profiles of distinct cell types less distinguishable, weakening the sensitivity of RNA-seq technology. Other experimental approaches, including cell purification and enrichment, are comparatively expensive and laborious [9]. Therefore developing alternative *in silico *approaches to resolving the tissue heterogeneity problem, especially in cancer research, remains a major problem in RNA-seq analysis [10].

Research in computational approaches to resolving the tissue heterogeneity problem of different biotechnologies has a fairly long history [11-14]. The first attempt to computationally micro-dissect heterogeneous tissues for microarray expression data was based on a linear model [11], which estimated both cell-type proportion and gene expression level. Prior information regarding "marker genes", which are genes uniquely expressed in each cell-type, was incorporated into the linear model to identify distinct cell types. The linear model was extended with Bayesian prior densities of cell-type proportions [13], and a posterior sampling approach was then constructed for cell-type-specific expression profiling. A statistical testing method [14] was proposed for single nucleotide polymorphism (SNP) array based copy number alterations analysis from heterogeneous tissue samples. In this method, Bayesian differentiation between hemizygous deletion and homozygous deletion were used to infer the underlying normal cell proportion and copy number profiles of both normal cells and tumor cells. One common feature shared by these methods is that they all adopted probabilistic models, not only allowing prior information about different cell types to be smoothly incorporated into the models, but also taking advantages of the flexibility of probabilistic model to capture specific aspects of each data type.

To the best of our knowledge, no computational approaches have been proposed to resolve the tissue heterogeneity problem from RNA-seq data in a probabilistic fashion. Typically, researchers apply transcriptional profiling tools designed for homogeneous tissue samples directly to RNA-seq data from heterogeneous tissue samples. Subsequent estimation results are interpreted as transcriptional profiling of a particular single cell type of interest. Therefore, we ask whether it is possible to estimate trancriptive abundances of individual cell types from RNA-seq of heterogeneous tissues, by decoupling the contributions from multiple cell types. We propose a probabilistic model-based approach, Transcript Estimation from Mixed Tissue samples (TEMT) to address this question. Currently, TEMT requires two sets of single-end RNA-seq reads. One read set is from a heterogeneous tissue sample composed of two cell types, while the other is from a pure tissue sample composed of one of the two cell types. TEMT incorporates prior information of cell type proportion and can calculate probabilities of RNA-seq reads sampled from each cell type. Because TEMT implements an online EM algorithm [15], it has a time requirement proportional to the data size and a constant memory requirement. To further improve the estimation accuracy, TEMT also implements a bias module, which incorporates both positional bias [16-18] and sequence-specific bias [19,20].

To assess the performance of TEMT, we analyzed a series of both simulation and real data from ENCODE [21], and compared the transcript relative abundances estimation from TEMT to those obtained from other methods that do not take the tissue heterogeneity into account. Our results show that explicitly accounting for tissue heterogeneity can significantly improve transcript abundance estimation accuracy.

## Methods

In this section, we first introduce the generative mixture model of TEMT. Combined with cell type proportion as prior information, we propose a maximum a posteriori estimation approach for finding model parameters. Next, we explain how to incorporate a positional and sequence-specific bias module into the model. Finally, we introduce an online EM algorithm for parameter estimation, reducing the time complexity to be proportional to the data size and the space complexity to be constant.

### Model

#### Basic definition

We focus on transcript abundance estimation. Denote T  as a set of reference transcripts, which we assume is known and complete. Let $l_{t}$ denote the length of transcript *t *in the set with t=1,⋯,T, where *T *is the total number of transcripts in the reference set. Suppose we are interested in transcriptome analysis in two cell types: *a *and *b*. Let $\rho_{t}^{a}$ and $\rho_{t}^{b}$ denote the relative transcript abundance of transcript *t *in cell type *a *and *b*, respectively, with t=1,⋯,T. We assume ${\left\{\rho_{t}^{a}\right\}}_{t=1}^{T}$ are ${\left\{\rho_{t}^{b}\right\}}_{t=1}^{T}$ properly normalized such that $\sum_{t=1}^{T}\rho_{t}^{a}=1$ and $\sum_{t=1}^{T}\rho_{t}^{b}=1$.

We assume RNA-seq reads are available in two samples: one consisting of cells of only type *a*, which we call the "pure sample", and the other consisting of cells of both type *a *and *b *with percentage $T^{a}$ from cell type *a *and $T^{b}$ from cell type *b*, which we call the "mixed sample." In the cancer transcriptome analysis, cell type *a *can represent normal cells as it is usually easy to obtain a pure tissue sample, while cell type *b *can represent tumor cells as most tumor tissue samples are contaminated by normal cells.

Because the pure sample consists of only cell type *a*, its relative transcript abundance $\rho_{t}^{p}$ is described by $\rho_{t}^{p}=\rho_{t}^{a}$ for all *t*. However, the relative abundance of transcript *t *within the mixed sample is a weighted sum of the transcript abundance of both cell type *a *and *b*

(1) $$\rho_{t}^{m}=\tau^{a}\rho_{t}^{a}+\tau^{b}\rho_{t}^{b},\tau_{t}^{a}+\tau_{t}^{b}=1$$

Denote the read set from the pure sample by $R^{p}$ and the read set from the mixed sample by$R^{m}$. Our goal is to estimate the relative abundance of each transcript in the reference set *T *from the RNA-seq read data $R^{p}$ and $R^{m}$ in both cell type *a *and *b*

### Alignment representation

We first map reads to the reference transcript set T  and convert the raw read data into a corresponding alignment representation. Denote the alignment representation of the read set $R^{p}$ by $Y^{p}$ =$\left\{y_{i,t}^{p}|i=1,\;\cdots \;,\;N^{p},\;t=1,\;\cdots \;,\;T\right\}$, where $y_{i,t}^{p}=1$ if read i from $R^{p}$ aligns to transcript *t *and 0 otherwise, and *N^p ^*is the total number of reads in read set $R^{p}$. The alignment representation $Y^{m}=\left\{y_{i,t}^{m}|i=1,\;\cdots \;,\;N^{m},\;t=1,\;\cdots \;,\;T\right\}$ is similarly defined for read set $R^{m}$ from the mixed sample. Note that one read might map to multiple transcripts due to alternative splicing, sequence similarity shared by homologous genes, or other reasons. As a result, the summation of $y_{i,t}^{p}$ over all transcripts may be bigger than 1 for some *i*. These "ambiguous reads" introduce a major source of uncertainty into transcript abundance estimation.

### Generative model

We model the sequencing of reads as a sampling process, randomly chooses a transcript *t *from the reference transcript set T  according to its relative abundance and effective length, and then generates a read from a random location of the chosen transcript. Under this model, the probability of a read originating from transcript *t *is

(2) $$\alpha_{t}^{s}=\frac{\rho_{t}^{s}{\tilde{l}}_{t}}{\sum_{k=1}^{T}\rho_{k}^{s}{\tilde{l}}_{k}}$$

with *s *being either *p *for the pure sample or *m *for the mixed sample. Here, ${\tilde{l}}_{t}$ is the effective length of transcript *t*, which quantifies the number of positions at which a read can start within transcript *t*. Different methods have been proposed to model the effective length [19,22]. In TEMT, the effective length is modeled with consideration to the length distribution of RNA-seq fragments [19]

(3) $${\tilde{l}}_{t}=\overset{l_{t}}{\underset{x=1}{\sum }}\frac{\phi \left(x;\mu ,\sigma^{2}\right)}{\overset{l_{t}}{\underset{x^{\prime }=1}{\sum }}\phi \left(x^{\prime };\mu ,\sigma^{2}\right)}\left(l_{t}-x+1\right)$$

We assume the fragment length *x *has a normal distribution with mean *µ *and variance $\sigma^{2}$, and $\phi \left(x;\mu ,\;\sigma^{2}\right)$ is the normal probability density function of. By renormalizing $\phi \left(x;\mu ,\;\sigma^{2}\right)$, we obtain the discrete distribution of all possible fragment lengths. The effective length ${\tilde{l}}_{t}$ is then the expectation of the number of positions a read can start within transcript *t*, based on the discrete distribution of fragment length.

Suppose a read is generated uniformly from each location covered by the effective length of each transcript. Then the probability of observing read *i *as represented by its alignment map is

(4) $$\mathbb{P}\left({\left\{y_{i,t}^{s}\right\}}_{t=1}^{T}\right)=\overset{T}{\underset{t=1}{\sum }}y_{i,t}^{s}\frac{\alpha_{t}^{s}}{{\tilde{l}}_{t}}$$

for *s *= *p *or *m*.

Assume each read is generated independently in both the pure and the mixed samples. The likelihood of observing the read set $R^{p}$from the pure sample and $R^{m}$ from the mixed sample is then described by

(5) $$\mathbb{P}\left(R^{p},\;R^{m}|{\left\{\alpha_{t}^{p}\right\}}_{t=1}^{T},\;{\left\{\alpha_{t}^{m}\right\}}_{t=1}^{T}\right)=\overset{N^{p}}{\underset{i=1}{\prod }}\overset{T}{\underset{t=1}{\sum }}y_{i,t}^{p}\frac{\alpha_{t}^{p}}{{\tilde{l}}_{t}}\overset{N^{m}}{\underset{i=1}{\prod }}\overset{T}{\underset{t=1}{\sum }}y_{i,t}^{m}\frac{\alpha_{t}^{m}}{{\tilde{l}}_{t}}$$

We are interested in estimating the relative transcript abundances set ${\left\{\rho_{t}^{a}\right\}}_{t=1}^{T}$, ${\left\{\rho_{t}^{b}\right\}}_{t=1}^{T}$ but since it can be uniquely defined by the read sampling probability set ${\left\{\alpha_{t}^{a}\right\}}_{t=1}^{T}$, ${\left\{\alpha_{t}^{b}\right\}}_{t=1}^{T}$

(6) $$\rho_{t}^{a}=\frac{\frac{\alpha_{t}^{a}}{\tilde{l_{t}}}}{\sum_{k=1}^{T}\frac{\alpha_{k}^{a}}{\tilde{l_{k}}}},\rho_{t}^{b}=\frac{\frac{\alpha_{t}^{b}}{\tilde{l_{t}}}}{\sum_{k=1}^{T}\frac{\alpha_{k}^{b}}{\tilde{l_{k}}}}$$

We can directly estimate the read sampling probability ${\left\{\alpha_{t}^{a}\right\}}_{t=1}^{T},{\left\{\alpha_{t}^{b}\right\}}_{t=1}^{T}$ set from the likelihood function Equation (5) instead. Note that, again $\alpha_{t}^{p}=\alpha_{t}^{a}$ for all *t *as it is the parameter of pure sample, but unlike the linear form in Equation (1), $\alpha_{t}^{m}$ in terms of $\alpha_{t}^{a},\alpha_{t}^{b}$ is given as a nonlinear form

(7) $$\alpha_{t}^{m}=\Lambda^{a}\tau^{a}\alpha_{t}^{a}+\Lambda^{b}\tau^{b}\alpha_{t}^{b}$$

(8) $$\Lambda^{a}=\frac{\sum_{k=1}^{T}\rho_{k}^{a}{\tilde{l}}_{k}}{\sum_{k=1}^{T}\rho_{k}^{m}{\tilde{l}}_{k}},\Lambda^{b}=\frac{\sum_{k=1}^{T}\rho_{k}^{b}{\tilde{l}}_{k}}{\sum_{k=1}^{T}\rho_{k}^{m}{\tilde{l}}_{k}}$$

Where, the factor $\Lambda^{a},\Lambda^{b}$ induce the nonlinearity. But due to the averaging effect of the large number of transcripts, practically $\Lambda^{a},\Lambda^{b}$ lies within 1 *± *0*:*05. So we approximate $\alpha_{t}^{m}$ with the linear form

(9) $$\alpha_{t}^{m}\approx \tau^{a}\alpha_{t}^{a}+\tau^{b}\alpha_{t}^{b}$$

As it brings computational convenience in the following learning step.

Finally, we define

(10) $$\text{Θ}=\left\{{\left\{\alpha_{t}^{a}\right\}}_{t=1}^{T},\;{\left\{\alpha_{t}^{b}\right\}}_{t=1}^{T},\;\tau^{a},\;\tau^{b}\right\}$$

as the parameters of our model. The likelihood in Equation (5) can be then expressed as

(11) $$\mathbb{P}\left(R^{p},\;R^{m}|\text{Θ}\right)=\overset{N^{p}}{\underset{i=1}{\prod }}\overset{T}{\underset{t=1}{\sum }}y_{i,t}^{p}\frac{\alpha_{t}^{a}}{{\tilde{l}}_{t}}\overset{N^{m}}{\underset{i=1}{\prod }}\overset{T}{\underset{t=1}{\sum }}y_{i,t}^{m}\frac{\left(\tau^{a}\alpha_{t}^{a}+\tau^{b}\alpha_{t}^{b}\right)}{{\tilde{l}}_{t}}$$

### Maximum a posteriori estimation

Several analysis have noticed the identifiability problem [12,13] in estimating cell type specific expression in heterogeneous tissue samples. Ideally, if the proportion information for some cell types is missing, we can then pool these cell types as one type, making the expression of each individual cell type inside unidentifiable. Previously, prior constraints have been used to resolve the problem [12,13]. In our model, the prior knowledge of cell type proportions is combined with the model likelihood, and we subsequently use maximum a posteriori (MAP) estimation to find the optimal parameters.

Specifically, we place a *Beta*$\left(\beta^{a},\;\beta^{b}\right)$ distribution as the prior for cell proportions of type *a *and type *b*. The parameter $\beta^{a},\;\beta^{b}$ quantify the location and sharpness of the prior. Practically, we found setting $\beta^{a},\;\beta^{b}$ 10 times as the data size gave a good convergence rate and accuracy. Combining the prior with the likelihood given in Equation (11), the posterior distribution of the model is proportional to

(12) $$\mathbb{P}\left(\Theta |R^{p},\;R^{m}\right)\propto \left(\overset{N^{p}}{\underset{i=1}{\prod }}\overset{T}{\underset{t=1}{\sum }}y_{i,t}^{p}\frac{\alpha_{t}^{a}}{{\tilde{l}}_{t}}\right)\;\;\left[\overset{N^{m}}{\underset{i=1}{\prod }}\overset{T}{\underset{t=1}{\sum }}y_{i,t}^{m}\frac{\left(\tau^{a}\alpha_{t}^{a}+\tau^{b}\alpha_{t}^{b}\right)}{{\tilde{l}}_{t}}\right]{\left(\tau^{a}\right)}^{\beta^{a}-1}{\left(\tau^{b}\right)}^{\beta^{b}-1}$$

### Incorporating sequencing bias

Both positional [16-18] and sequence-specific [19,20] sequencing biases have been observed in next generation sequencing data. These biases mainly result from non-uniformly distributed cDNA fragments during the RNA-seq library preparation [20]. Under positional bias, reads positioning is not uniformly distributed across the effective length of the target transcript, but preferentially distributed around either the 5' end or the 3' end of the target transcript. Under sequence-specific bias, the sequences near the two ends of the fragments affect their probability to be sequenced. To account for these non-uniformity effects during transcript abundance estimation, we incorporate the bias module of [19] into our model.

In order to further describe the local alignment context, we define another two sets of variables. Specifically, for read *i *from either read set $R^{p}$ or $R^{m}$, we denote $b_{i,\;\;t}^{s}\in \left[0,\;\;{\tilde{l}}_{t}\right]$ as the starting position of the alignment within transcript *t *relative to the 5' end of the strand. We also denote $\pi_{i,\;\;t}^{s}\in \Sigma^{L}$, where Σ={A,C,G,T}, as the local sequence of transcript *t *with length *L *and centered at $b_{i,\;t}^{s}$.Then we define the bias weight $w_{i,\;t}^{s}$ as

(13) $$w_{i,t}^{s}=\frac{\mathbb{P}\left(b_{i,\;t}^{s}|\text{bias}\right)\mathbb{P}\left(\pi_{i,\;t}^{s}|\text{bias}\right)}{\mathbb{P}\left(b_{i,\;t}^{s}|\text{uniform}\right)\mathbb{P}\left(\pi_{i,\;t}^{s}|\text{uniform}\right)}$$

for s=*p *or *m*.

This bias weight $w_{i,\;t}^{s}$ is essentially the ratio of the probability of observing $b_{i,\;\;t}^{s}$*and *$\pi_{i,\;t}^{s}$ under the bias model to the probability under the uniform model. If no bias exists, the weight $w_{i,\;t}^{s}$ reduces to 1. The bias re-weighted Equation (4) is then:

(14) $$\mathbb{P}\left({\left\{y_{i,\;t}^{s}\right\}}_{t\;=\;1}^{T}\right)=\overset{T}{\underset{t\;=\;1}{\sum }}y_{i,\;t}^{s}\frac{\alpha_{t}^{s}}{{\tilde{l}}_{t}}w_{i,\;t}^{s}$$

To calculate the bias weight, we use the bin method and Markov chain for positional bias and sequence-specific bias respectively. Complete details can be found in the Supplementary (Additional file 1). The final unnormalized posterior distribution of the model is then described as

(15) $$\mathbb{P}\left(\Theta |R^{p},\;R^{m}\right)\propto \left(\overset{N^{p}}{\underset{i\;=\;1}{\prod }}\overset{T}{\underset{t\;=\;1}{\sum }}y_{i,\;t}^{p}\frac{\alpha_{t}^{a}}{{\tilde{l}}_{t}}w_{i,\;t}^{p}\right)\;\left[\overset{N^{m}}{\underset{i=1}{\prod }}\overset{T}{\underset{t=1}{\sum }}y_{i,\;t}^{m}\frac{\left(\tau^{a}\alpha_{t}^{a}+\tau^{b}\alpha_{t}^{b}\right)}{{\tilde{l}}_{t}}w_{i,\;t}^{m}\right]\;{\left(\tau^{a}\right)}^{\beta^{a}-1}{\left(\tau^{b}\right)}^{\beta^{b}-1}$$

**Figure 1:**
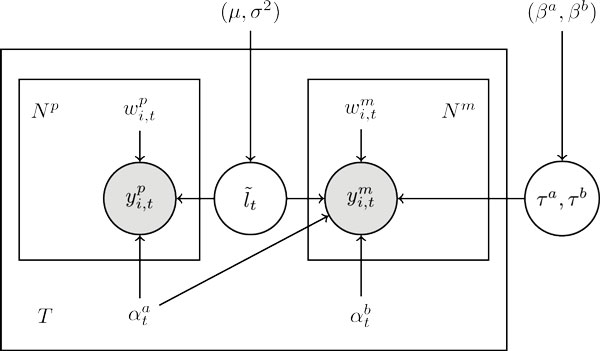
**The representative graphical model of TEMT**.

Where $w_{i,\;t}^{p}$ and $w_{i,\;t}^{m}$ are the bias weights computed based on read set $R^{p}$ and $R^{m}$. The directed graphical model of TEMT is shown in Figure 1. The estimated parameters are given by

(16) $$\hat{\Theta }=\underset{\theta }{\text{argmax}}\text{log}\mathbb{P}\left(\Theta |R^{p},\;R^{m}\right)$$

### Online EM algorithm for learning

We solve the maximum a posteriori problem in Equation (16) using the Expectation-Maximization (EM) [23] framework. For each read *i *from read set $R^{p}$ of pure sample, we denote the latent variable of the transcript alignment representation as $Z_{i}^{p}=\left\{z_{i,t}^{p}|t=1,\;\cdots \;,\;T\right\}$, where $z_{i,t}^{p}=1$if read *i *aligns to transcript *t *and 0 otherwise. But now $\overset{T}{\underset{t=1}{\sum }}z_{i,t}^{p}=1$, which means only one $z_{i,t}^{p}=1$, indicating read *i *is actually originating from transcript *t*. Similarly, for each read *i *from read set $R^{m}$ of mixed sample, we denote the latent variable of the transcript alignment representation as $Z_{i}^{m}=\left\{z_{i,t}^{ma},\;z_{i,t}^{mb}|t=1,\;\cdots \;,\;T\right\}$, where $z_{i,t}^{ma}=1$ if read *i *aligns to transcript *t *and is originating from cell type *a *within the mixed sample, and 0 otherwise. $z_{i,t}^{mb}=1$ or 0 is similar defined for cell type *b*. Thus $\sum_{t=1}^{T}\left(z_{i,\;t}^{ma}+z_{i,\;t}^{mb}\right)=1$ means read *i *is actually originating from only one transcript, and either from cell type *a *or *b *within the mixed sample. We also define the auxiliary variable $q_{i,t}^{p}=\mathbb{P}\left(z_{i,t}^{p}=1|\Theta ,\;Y^{p},\;Y^{m}\right)$, $q_{i,\;t}^{ma}=\mathbb{P}\left(z_{i,\;t}^{ma}=1|\Theta ,\;Y^{p},\;Y^{m}\right)$ and $q_{i,\;t}^{mb}=\mathbb{P}\left(z_{i,\;t}^{mb}=1|\Theta ,\;Y^{p},\;Y^{m}\right)$ as the conditional probability weight of each latent variable $z_{i,\;t}^{p}=1$, $z_{i,\;t}^{ma}=1$ and $z_{i,\;t}^{mb}=1$ conditional on model parameters Θ and the observed read alignment representations $Y^{p}Y^{m}$. Then based on Jensen's inequality [24], the complete posterior distribution, which is also the lower bound of Equation (15) can be written as

(17) $$\mathbb{P}\left(\Theta |R^{p},\;R^{m}\right)\geq \frac{1}{C}\left[\overset{N^{p}}{\underset{i=1}{\prod }}\overset{T}{\underset{t=1}{\prod }}{\left(\frac{\alpha_{t}^{a}}{{\tilde{l}}_{t}}w_{i,t}^{p}\right)}^{q_{i,\;t}^{p}}\right]\;\left[\overset{N^{m}}{\underset{i=1}{\prod }}\overset{T}{\underset{t=1}{\prod }}{\left(\frac{\tau^{a}\alpha_{t}^{a}}{{\tilde{l}}_{t}}w_{i,t}^{m}\right)}^{q_{i,\;t}^{ma}}{\left(\frac{\tau^{b}\alpha_{t}^{b}}{{\tilde{l}}_{t}}w_{i,t}^{m}\right)}^{q_{it}^{mb}}\right]{\left(\tau^{a}\right)}^{\beta^{a}-1}{\left(\tau^{b}\right)}^{\beta^{b}-1}$$

In which *C *is a normalizing constant and the equality holds only if the conditional probabilities $q_{i,\;t}^{p}$, $q_{i,\;t}^{ma}$, $q_{i,\;t}^{mb}$ are the true posterior distributions of latent variables ${\left\{Z_{i}^{p}\right\}}_{i=1}^{N^{p}},{\left\{Z_{i}^{m}\right\}}_{i=1}^{N^{m}}$.

The EM framework maximizes Equation (17) by iteratively applying the expectation step and the maximization step to update both the conditional probabilities $q_{i,\;t}^{p}$, $q_{i,\;t}^{ma}$, $q_{i,\;t}^{mb}$ and model parameters Θ until convergence. The expectation step of typical batch EM algorithm has to fetch all the data points into memory, and calculates the conditional probabilities based on the average of all the data points. While this batch method guarantee's the log-likelihood function to monotonically increase, it also induces inefficiency in both time and space complexity. Considering the high-throughput nature of next-generation sequencing technology as well as its huge data size, we implemented the EM algorithm in an online fashion [15] to both lower the memory requirement and boost the convergence rate.

The main difference between the batch EM and the online EM is in the E-step. The E-step of the online EM algorithm first calculates the conditional probabilities of only one new data point, and then updates the conditional probabilities of all the current data points by interpolating between the conditional probabilities of all the previous data points and the conditional probabilities of the new data point, with a forgetting factor *σ *controlling the convergence rate.

It is shown in [15] that with the constraint 0.5 *< σ *≤ 1, the online EM algorithm is asymptotically equivalent to stochastic gradient ascent, and is guaranteed to converge to the maximum likelihood estimator, which is extended to the maximum a posteriori estimator in our model.

Specifically, the online EM updates in our model is given by

### E-step

(18) $$q_{i+1,\;t}^{p}=\frac{y_{i+1,\;t}^{p}\frac{\alpha_{t}^{a(n)}}{{\tilde{l}}_{t}}w_{i,\;t}^{p}}{{\sum^{\text{​}}}_{k=1}^{T}y_{i+1,\;k}^{p}\frac{\alpha_{k}^{a(n)})}{{\tilde{l}}_{k}}w_{i,\;k}^{p}}$$

(19) $$q_{i+1,\;t}^{ma}=\frac{y_{i+1,\;t}^{m}\frac{\tau^{a\left(n\right)}\alpha_{t}^{a\left(n\right)}}{{\tilde{l}}_{t}}w_{i,\;t}^{m}}{\sum_{k=1}^{T}y_{i+1,\;k}^{m}\frac{\tau^{a\left(n\right)}\alpha_{k}^{a\left(n\right)}+\tau^{b\left(n\right)}\alpha_{k}^{b\left(n\right)}}{{\tilde{l}}_{t}}w_{i,\;k}^{m}}$$

(20) $$q_{i+1,\;t}^{mb}=\frac{y_{i+1,\;t}^{m}\frac{\tau^{b\left(n\right)}\alpha_{t}^{b\left(n\right)}}{{\tilde{l}}_{t}}w_{i,\;t}^{m}}{\sum_{k=1}^{T}y_{i+1,\;k}^{m}\frac{\tau^{a\left(n\right)}\alpha_{k}^{a\left(n\right)}+\tau^{b\left(n\right)}\alpha_{k}^{b\left(n\right)}}{{\tilde{l}}_{k}}w_{i,\;k}^{m}}$$

(21) $$q_{*,\;t}^{p\left(n+1\right)}=\left[1-\frac{1}{{\left(n+2\right)}^{\sigma }}\right]\;q_{*,\;t}^{p\left(n\right)}+\frac{1}{{\left(n+2\right)}^{\sigma }}q_{i+\text{i},\;t}^{p}$$

(22) $$q_{*,t}^{ma\left(n+1\right)}=\left[1-\frac{1}{{\left(n+2\right)}^{\sigma }}\right]q_{*,\;t}^{ma\left(n\right)}+\frac{1}{{\left(n+2\right)}^{\sigma }}q_{i+\text{i},\;t}^{ma}$$

(23) $$q_{*,\;t}^{mb\left(n+1\right)}=\left[1-\frac{1}{{\left(n+2\right)}^{\sigma }}\right]q_{*,\;t}^{mb\left(n\right)}+\frac{1}{{\left(n+2\right)}^{\sigma }}q_{i+\text{i},\;t}^{mb}$$

In Equation (18-20), we compute the conditional probabilities $q_{i+1,\;t}^{p}$, $q_{i+1,\;t}^{ma}$, $q_{i+1,\;t}^{mb}$ of just one new read *i *+ 1 based on previous parameter estimation ${\left\{\alpha_{t}^{a\left(n\right)}\right\}}_{t\;=\;1}^{T}$, ${\left\{\alpha_{t}^{b\left(n\right)}\right\}}_{t\;=\;1}^{T}$, $\tau^{a\left(n\right)},\tau^{b\left(n\right)}$; In Equation (21-23), we compute the new conditional probabilities average $q_{*,\;t}^{p\left(n+1\right)}$, $q_{*,\;t}^{ma\left(n+1\right)}$, $q_{*,\;t}^{mb\left(n+1\right)}$ by interpolating between the previous conditional probabilities average $q_{*,\;t}^{p\left(n\right)}$, $q_{*,\;t}^{ma\left(n\right)}$, $q_{*,\;t}^{mb\left(n\right)}$ and $q_{i+1,\;t}^{p}$, $q_{i+1,\;t}^{ma}$, $q_{i+1,\;t}^{mb}$. *n *is the index of iteration step and *i *is the index of data points. *σ *is the forgetting factor which controls the convergence rate, with the constraint 0.5 *< σ *≤ 1.

### M-step

(24) $$\tau^{a(n+1)}=\frac{{\sum^{\text{​}}}_{t=1}^{T}q_{*,t}^{ma(n+1)}+\frac{\beta^{a}-1}{N^{m}}}{1+\frac{\beta^{a}+\beta^{b}-2}{N^{m}}}$$

(25) $$\tau^{b(n+1)}=\frac{{\sum^{\text{​}}}_{t=1}^{T}q_{*,t}^{mb(n+1)}+\frac{\beta^{b}-1}{N^{m}}}{1+\frac{\beta^{a}+\beta^{b}-2}{N^{m}}}$$

(26) $$\alpha_{t}^{a(n+1)}=\frac{q_{*,t}^{p(n+1)}+q_{*,t}^{ma(n+1)}}{1+\tau^{a(n+1)}}$$

(27) $$\alpha_{t}^{b(n+1)}=\frac{q_{*,t}^{mb(n+1)}}{\tau^{b(n+1)}}$$

In the subsequent M-step, parameters ${\left\{\alpha_{t}^{a\left(n+1\right)}\right\}}_{t\;=\;1}^{T}$, ${\left\{\alpha_{t}^{b\left(n+1\right)}\right\}}_{t\;=\;1}^{T}$, $\tau^{a\left(n+1\right)}$, $\tau^{b\left(n+1\right)}$ are updated according to new conditional probabilities average $q_{*,\;\;t}^{p\left(n+1\right)}$, $q_{*,\;\;t}^{ma\left(n+1\right)}$, $q_{*,\;\;t}^{mb\left(n+1\right)}$.

## Results

Next we test the performance of the proposed method on both simulation data and the recently released ENCODE data [21]. For both datasets, we used the following three-step protocol and parameters to construct the analysis:

**1**. We aligned the raw read set from either simulation or the ENCODE data to a given transcript set using bowtie-0.12.7 [25]. For each read, we allowed 2 mismatches and reported at most 10 candidate alignments.

**2**. The abundance of each transcript in terms of estimated counts was estimated via both TEMT and a control model. Estimated counts is defined as the estimated number of reads generated from the target transcript. In TEMT, the prior of each cell type proportion was set to the same as the proportion used in simulation and ENCODE data respectively, and *β^a^*, *β^b ^*was set to 10 times the size of the read set $R^{m}$. *μ *= 200; *σ *= 80 were used as the mean and standard deviation of the RNA-seq fragment length distribution. We chose eXpress-0.9.4 [26] as the control model, as it is the state-of-the-art method for transcript abundance estimation and also utilizes an online-EM algorithm. Note that, to run TEMT, we need two read sets, in which one is for the pure sample and the other is for the mixed sample, as previously mentioned. In contrast, to run eXpress, we only need one read set from either the pure sample or the mixed sample. The forgetting factor for the on-line EM algorithms in both TEMT and eXpress was set to be *σ *= 0*:*85, and the error-model in eXpress was disabled for comparison.

**3**. To measure the model accuracy, we used the Error Fraction (EF) measure introduced by [17] to quantify the discrepancy between the model estimates and the ground truth estimates. The Error Fraction is defined as the fraction of transcripts for which the estimates are significantly different (percent error *>*10% in our case) from the ground truth.

### Simulation

#### Data preparation

To show the utility of TEMT, we first carried out a series of simulation studies. To obtain simulated read sets, we used FluxSimulator [27], a software for transcriptome and read generation by simulating the biochemical processes underlying the library preparation. FluxSimulator requires a reference transcript set to start the simulation process, so we manually downloaded 406 transcripts of 208 alternatively spliced genes in human from Alternative Splicing Structural Genomics Project (AS3D) [28], and used these 406 transcripts as the reference transcript set. We first simulated the transcript expression process twice producing two sets of relative transcript abundances, corresponding to cell type a and b respectively. Based on these two transcript abundance sets, we then simulated 6 pairs of 1 million 75-bp single-end read sets corresponding to six different cell type *b *proportions from 40% up to 90%. The relative transcript abundances of cell type *a *and *b *were kept the same throughout these simulations. For each paired read set, one read set is for the pure sample composed of only cell type *a*, whereas the other read set is for the mixed sample composed of both cell type *a *and *b*, mixed with the cell type *b *proportion. Within the mixed-sample read set, we also extracted the reads simulated purely from cell type *b*, which was used for control model eXpress.

#### Analysis

**Figure 2:**
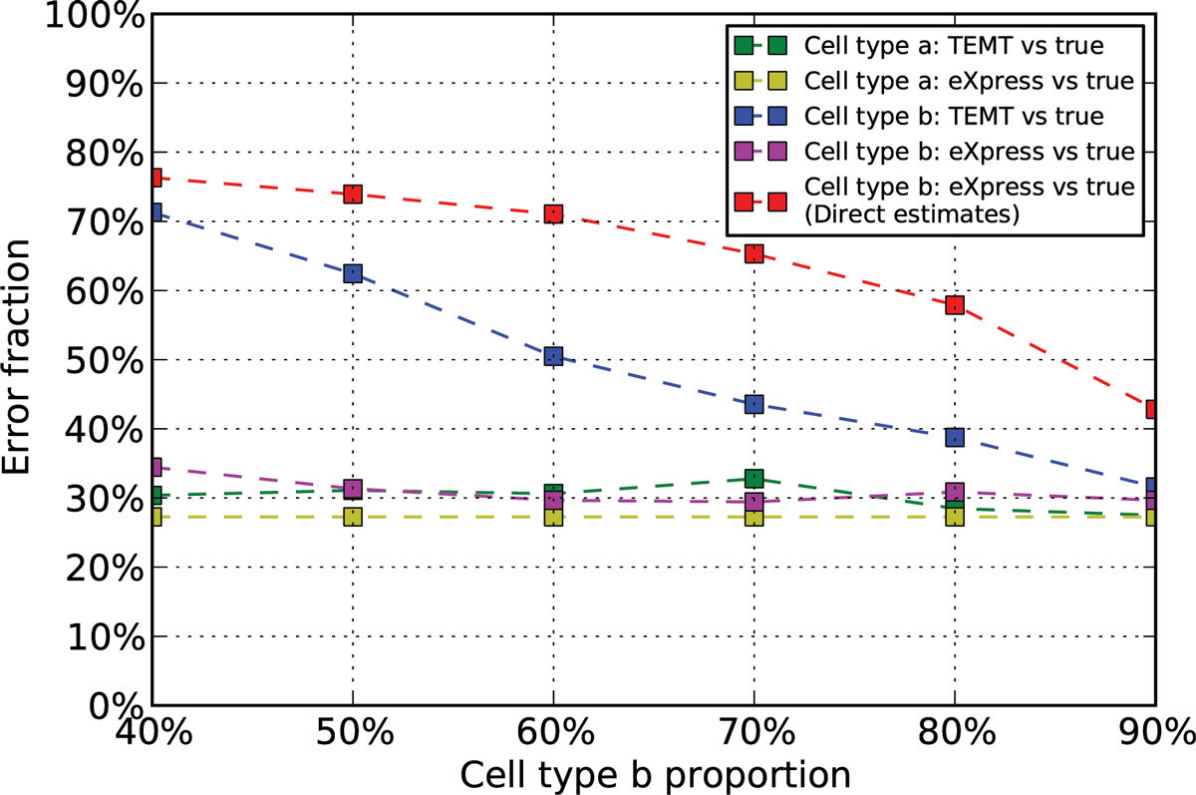
**Analysis results of simulated data of 6 different cell type ***b ***proportions with the bias module disabled**. The x-axis is the different cell type b proportion, and the y-axis is the Error Fraction of the corresponding estimates. The green and blue lines are the estimates from TEMT for cell type *a *and cell type *b*, based on the two read sets of the cell type *a *pure sample and the mixed sample. The yellow and magenta lines are the estimates from eXpress for cell type *a *and cell type *b*, based on the two read sets of the cell type *a *pure sample and the cell type *b *pure sample. The red line is the direct estimates from eXpress for cell type *b*, based on the read set of the mixed sample.

The simulated data are analyzed with the bias module both enabled and disabled. Surprisingly, the positional and sequence-specific bias module did not improve the accuracy of the transcript abundance estimation as measured by the Error Fraction of estimated counts in both TEMT and eXpress. This result may due to the stochasticity during the simulation of FluxSimulator. So we only present the results with the bias module disabled in both TEMP and eXpress in Figure 2.

We note that the estimates of cell type *a *from TEMT achieve roughly the same accuracy, compared with the estimates from eXpress based on the read set of the pure sample of cell type *a*. Also, this accuracy does not change significantly under the effect of different cell type b proportions. This is mainly due to the pure sample read set of cell type *a *within the input data for TEMT.

**Figure 3:**
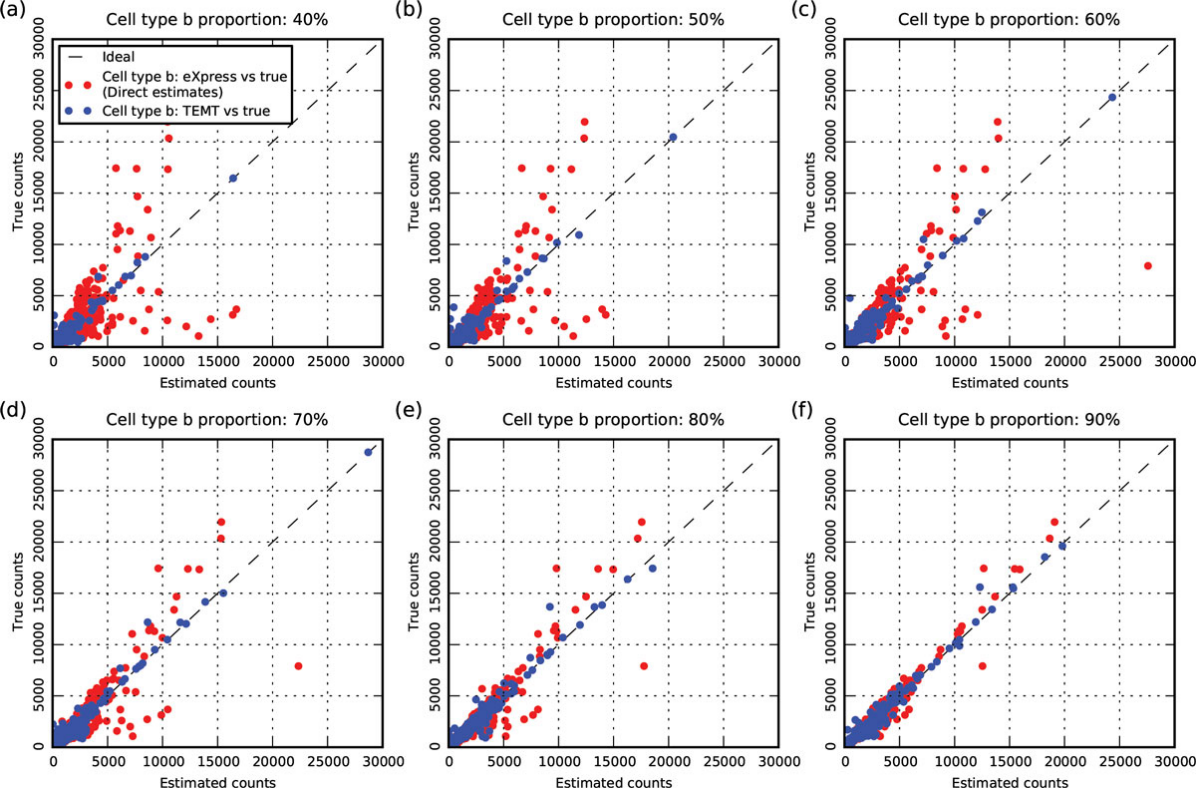
**Comparisons between indirect estimates from TEMT and direct estimates from eXpress for cell type ***b ***in terms of estimated counts**. The x-axis is the estimated counts from the two models, and the y-axis is the true counts. Each point in the figure is a comparison between the estimated count and true count. The red points are the direct estimates from eXpress, while the blue points are the indirect estimates from TEMT. Figure (**a**)-(**f**) are each comparison with cell type *b *proportions from 40% to 90%.

The accuracy of the estimates of cell type *b *from TEMT is also shown in Figure 2, which shows that TEMT generally outperforms the direct estimation method. To the best of our knowledge, there are no computational tools similar to our model that can estimate the relative transcript abundances of cell type *b *via RNA-seq data generated from mixed samples. Typically, computational methods are applied directly to the noisy data of mixed samples and results are interpreted as the estimates of cell type *b*. To compare the estimates of cell type *b *from TEMT with direct estimates using the current method, we applied the control model eXpress directly to the read set of the mixed sample. The estimated counts from eXpress were then compared with the true counts from another 1 million simulated read set purely of cell type *b*, while keeping the same relative transcript abundance as the previous simulations. The corresponding Error Fractions are shown as the red line in Figure 2 regarding different cell type *b *proportions. Although the accuracy of cell type *b *estimates from TEMT is affected by different cell type *b *proportions, it is generally better than the direct estimates. This can be further illustrated in Figure 3, which shows that the direct estimated counts of cell type *b *from eXpress deviate more from the true counts as the cell type *b *proportion decrease, while the estimates of TEMT have much reduced deviation. We notice that as the cell type *b *proportion gradually decreases, the accuracy of the estimates of cell type *b *from TEMT also decreases. This is the result of the contamination effect from the cell type *a *within the mixed sample. A recent paper [4] also observed this similar phenomenon when studying copy number aberrations from heterogeneous tumor tissue.

### ENCODE data

#### Data preparation

Next we analyzed the recently released ENCODE data. Due to the lack of RNA-seq data sampled from mixed tissue samples with known cell type proportions, we artificially generated the mixed-sample read sets by mixing reads obtained from two different cell types. Specifically, we chose two Tier 1 cell lines, GM12878 and K562, and treated them as cell type *a *and cell type *b *respectively. The corresponding single-end RNA-seq data of these two cell lines, GM78 1×75D A 1 (UCSC Accession: wgEncodeEH000125) and K562 1×75D A 1 (UCSC Accession: wgEncodeEH000126) from the Wold lab [29] at Caltech, were download from ENCODE (2012). The data downloaded from the same lab under similar protocols is intended to reduce the deviation resulting from experiments. We then randomly selected 10 million reads from GM12878 cells to form the read set of the pure sample, and 10 million reads from both GM12878 and K562 cells using different K562 cells proportions to form the read set of the mixed sample. Similar to the previous simulation study, we extracted the reads purely selected from K562 cells within the mixed sample, and used them for the eXpress control model. We studied 6 different K562 cells proportions from 40% to 90% in order to compare with the previous simulation study. 36908 human RefSeq [30] transcripts from UCSC known genes [31] were used as the transcript set for the ENCODE data.

#### Analysis

**Figure 4:**
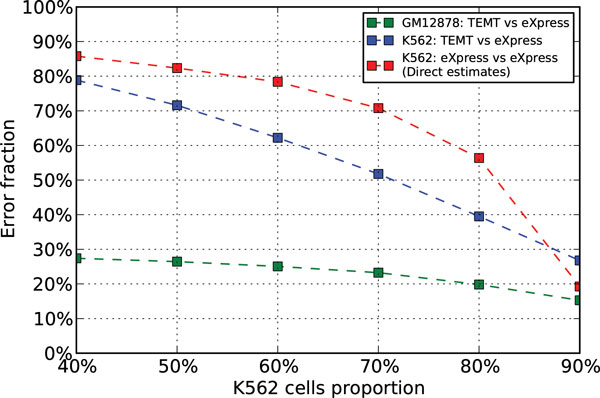
**Analysis results of the ENCODE data of 6 different K562 cells proportions with the bias module disabled**. The x-axis is the different K562 cells proportions, and the y-axis is the Error Fraction of the corresponding estimates. The green and blue lines are the estimates from TEMT for GM12878 and K562 cells, based on the read sets of the GM12878 cells pure sample and the mixed sample. The red line is the direct estimates from eXpress for K562 cells, based on the read set of the mixed sample.

One major issue in studying the ENCODE data is that the ground truth of relative transcript abundance in each cell type is unknown. We used the estimates from eXpress based on the GM12878 and K562 pure samples as the ground truth. Again, the bias module was disabled for both TEMT and eXpress. The general result of ENCODE data is shown in Figure 4. Similar to the simulated data, the indirect estimates for K562 cells from TEMT generally outperforms the direct estimates from eXpress based on the read set of the mixed sample. The contamination effect from cell type *a *within the mixed sample observed in Figure 3 is also seen in the eXpress analysis of ENCODE data, while TEMT does not have this issue. Note that the measure of relative transcript abundances as shown in the red line of Figure 4 is no longer estimated counts, but reads per kilobase of transcript per million mapped reads (RPKM), as the total number of reads from K562 cells within the mixed sample is less than the total number of reads of the mixed sample, so that normalization is necessary for comparison. We notice TEMT underperforms direct estimates from eXpress when K562 cells proportion equals 90%. Possibly the contamination effect of GM12878 cells within the mixed sample is not severe enough at this point, as we can imagine the red line in Figure 4 will finally reach 0% Error Fraction when K562 cells proportion reaches 100%. On the other hand, since the estimates from eXpress based on the pure sample are considered the ground truth, the lower bound Error Fraction of K562 cells estimates from TEMT should be the same as the Error Fraction of GM12878 cells estimates, which is around 20% to 30% in Figure 4.

## Discussion

We formulated our model under the assumption that the heterogeneous tissue is only composed of two cell types, but in reality, a heterogeneous tissue might be much more complicated, consisting of multiple cell types. To relax this constraint, our model needs to be further extended to analyze more complex cases in which each cell type may have its own subtypes, e.g. breast cancer subtypes, leading to a more sophisticated heterogeneous tissue environment. Further dissecting cell subtype heterogeneity is the next step in refining our model. Moving from two cell types to arbitrarily many cell types is of great interest, since it may substantially facilitate transcriptome study of heterogeneous tissues.

One critical component necessary to make our model work is the prior information of cell type *b *proportion, which is necessary to resolve the identifiability problem of mixed samples. In real experiments, precise prior information regarding cell type proportions may be unavailable. One solution in the context of our model is to down weight the effect of the prior by decreasing the parameter *β^a^*, *β^b^*, which adds more uncertainty to the cell mixture proportion. However, this approach may decrease the performance of the model as the uncertainty in cell mixture proportion cannot be distinguished from the uncertainty in transcript abundance estimation. This observation suggests another direction to further improving our model which is to solely estimate cell type *b *proportion without the prior information. To fulfill this requirement, the identifiability problem needs to be resolved as mentioned in section 2.3, which turns out to be comparatively hard for RNA-seq data. Unlike the heterozygous and homozygous deletions in [14], which can be utilized to differentiate between the SNP array data generated by normal cells and tumor cells, there are no such explicit differences between the reads generated by distinct cell types in RNA-seq data, thus making the generative mixture model unconstrained. The "marker genes" method proposed by [11], which tries to distinguish distinct cell types by utilizing genes uniquely expressed in each cell type, provides a future potential direction to extend the current model.

## Conclusion

In this article, we propose a probabilistic model-based method TEMT to estimate transcript abundance of individual cell types based on RNA-seq data from heterogeneous tissue samples. TEMT utilizes prior information to distinguish reads generated by each cell type within the heterogeneous tissue sample. Positional and sequence-specific biases are also incorporated to improve estimation accuracy. TEMT is able to process large datasets as the online EM algorithm is adopted to guarantee a time complexity proportional to the data size and a constant space complexity. Our experiments on both simulated datasets and ENCODE data shows that explicitly accounting for tissue heterogeneity can significantly improve the accuracy of transcript abundance estimation.

## Competing interests

The authors declare that they have no conflict of interests.

## Authors' contributions

Designed the experiments: LY and XX; Performed the experiments: LY; Wrote the paper: LY and XX; All authors contributed to the analysis, and approved the paper.

## Supplementary Material

Additional file 1 **Supplementary**. Complete details for calculating positional and sequence-specific bias weights. Click here for file

## Declarations

Publication of this article was supported by National Institute of Health grant R01HG006870.

This article has been published as part of *BMC Bioinformatics *Volume 14 Supplement 5, 2013: Proceedings of the Third Annual RECOMB Satellite Workshop on Massively Parallel Sequencing (RECOMB-seq 2013). The full contents of the supplement are available online at http://www.biomedcentral.com/bmcbioinformatics/supplements/14/S5.
