# Supplementary material for: A mixture model for expression deconvolution from RNA-seq in heterogeneous tissues
Source: BMC Bioinformatics. 2013 Apr 10;14(Suppl 5):S11. doi: 10.1186/1471-2105-14-S5-S11 (PMC3622628; doi:10.1186/1471-2105-14-S5-S11)
Supplement: Additional file 1 — Supplementary. Complete details for calculating positional and sequence-specific bias weights. [file 1471-2105-14-S5-S11-S1.pdf]

Supplementary

Bias weight calculation

To calculate the bias weight in Equation (13), we used the bin method and Markov chain for positional bias and sequence-specific bias respectively, which are described here.

Positional bias To capture the positional bias, we binned the relative positions within a transcript into 10 bins. The number of reads aligned to each bin were then counted for read set $\mathcal{R}^p$ and $\mathcal{R}^m$ respectively. If a read aligns to multiple transcripts, the count is then evenly divided and assigned to each transcript. It is reported in [1] that the positional bias is correlated to the transcript length, thus we further categorized the lengths of transcripts into 5 categories in Table 1, and added up the counts of each category. Denote $\mathcal{C}^p = \{c^p_{m,n} | m = 1, \cdots, 10, n = 1, \cdots, 5\}$ as the counts added up from $\mathcal{R}^p$, where $c^p_{m,n}$ is the number of reads aligned to bin m of transcript category n within read set $\mathcal{R}^p$. Thus, if the starting position $b^p_{i,t}$ of read i aligning to transcript t lies within bin m of transcript t and transcript t is in category n according to its length, the probability of observing $b^p_{i,t}$ under positional bias model is then given by:

$$\mathbb{P}(b^p_{i,t}|bias) = \frac{c^p_{m,n}}{\sum_{k=1}^{10} c^p_{k,n}} \tag{1}$$

While the probability of observing $b^p_{i,t}$ under uniform model is $\mathbb{P}(b^p_{i,t}|uniform) = \frac{1}{10}$, since the starting positions of reads are assumed to be uniformly distributed across the 10 bins of a transcript. The bias weight for read set $\mathcal{R}^m$ is similar defined.

Sequence specific bias We used a first order Markov chain to capture the sequence specific bias. Given the definition of $\pi^s_{i,t}$ in section 2.3, we used $L = 21$ as the window size. The probabilities $\mathbb{P}(\pi^s_{i,t}|bias), \mathbb{P}(\pi^s_{i,t}|uniform)$ in Equation (13) are given by:

$$\mathbb{P}(\pi^s_{i,t}|bias) = \mathbb{P}(\pi^s_{i,t,1}|bias) \prod_{j=2}^{21} \mathbb{P}(\pi^s_{i,t,j}|\pi^s_{i,t,j-1}, bias) \tag{2}$$

$$\mathbb{P}(\pi^s_{i,t}|uniform) = \mathbb{P}(\pi^s_{i,t,1}|uniform) \prod_{j=2}^{21} \mathbb{P}(\pi^s_{i,t,j}|\pi^s_{i,t,j-1}, uniform) \tag{3}$$

for $s = p$ or m.

$\pi^s_{i,t,j} \in \{A, C, G, T\}$ is the jth nucleotide of sequence $\pi^s_{i,t}$ regarding the alignment strand. $\mathbb{P}(\pi^s_{i,t,1}|bias)$ and $\mathbb{P}(\pi^s_{i,t,1}|uniform)$ are the empirical distribution of one nucleotide observed under the bias model and the uniform model respectively, while $\mathbb{P}(\pi^s_{i,t,j}|\pi^s_{i,t,j-1}, bias)$ and $\mathbb{P}(\pi^s_{i,t,j}|\pi^s_{i,t,j-1}, uniform)$ are the empirical conditional distributions of dinucleotide. The empirical distributions under the bias model were calculated based on the local sequences of all the alignments observed from read set $\mathcal{R}^p$ and $\mathcal{R}^m$ respectively. The

Table 1: The 5 categories of transcripts defined by their lengths.

Categories	Transcript Lengths (bp)
1	$1 \sim 791$
2	$792 \sim 1265$
3	$1266 \sim 1707$
4	$1708 \sim 2433$
5	$2434 \sim +\infty$

empirical distributions under the uniform model were calculated based on all the short sequences of window size 21 across the whole length of each transcript from the reference transcript set.

References

1. Bohnert R, Rätsch G: **rQuant. web: a tool for RNA-Seq-based transcript quantitation**. *Nucleic acids research* 2010, **38**(suppl 2):W348–W351.
